# Mediator Roles of Social Support and Hope in the Relationship Between Body Image Distress and Resilience in Breast Cancer Patients Undergoing Treatment: A Modeling Analysis

**DOI:** 10.3389/fpsyg.2021.695682

**Published:** 2021-09-24

**Authors:** Hsin-Tien Hsu, Chiung-Hui Juan, Jyu-Lin Chen, Hsiu-Fen Hsieh

**Affiliations:** ^1^School of Nursing, Kaohsiung Medical University, Kaohsiung, Taiwan; ^2^Department of Nursing, Kaohsiung Medical University Hospital, Kaohsiung Medical University, Kaohsiung, Taiwan; ^3^Department of Medical Research, Kaohsiung Medical University Hospital, Kaohsiung Medical University, Kaohsiung, Taiwan; ^4^Department of Family Health Care Nursing, University of California, San Francisco, San Francisco, CA, United States

**Keywords:** resilience, hope, social support, breast cancer, body image distress

## Abstract

Breast cancer and its treatment are particularly distressing for patients because of their potential impacts on body image. The most difficult phase of cancer treatment is usually the first year after a diagnosis. Cancer patients with strong resilience have the positive attitude, internal strength and external resources needed to cope with the disease and its treatment. This cross-sectional study investigated the mediator roles of hope and social support in the association between body image distress and resilience. A structured questionnaire was used to collect data for a convenience sample of 141 breast cancer patients undergoing treatment in southern Taiwan. Structural equation modeling was used for data analysis. The results showed that the final model had a good fit to the data and accounted for 51% of the total variance in resilience. The model of multiple parallel mediators of resilience revealed that hope and social support had mediator roles in the effect of body image distress on resilience. Hope had an important partial mediating role in the association between body image distress and resilience. Social support also had a partial mediating role in the relationship between body image distress and resilience. Social support did not directly affect resilience and indirectly affected resilience through hope. Psychosocial interventions aimed at reducing the impact of body image distress and increasing resilience in breast cancer patients should focus on cultivating hope and increasing social support, particularly support from family members and health professionals.

## Introduction

Breast cancer is the most common cancer in women worldwide (Gradishar et al., 2020). In Taiwan, the incidence of breast cancer in women exceeds that of all other cancer types (Hsu et al., 2017). Statistically, breast cancer is rare in women younger than 25 years, but the incidence increases with age until age 50 years and then plateaus at ages 50–69 years (Health Promotion Administration Ministry of Health Welfare, 2018). Current treatment for stage I-III breast cancer is mainly surgical treatment combined with adjuvant therapy. Stage IV breast cancer is mainly treated with systemic therapy, including chemotherapy, hormone therapy, targeted therapy, immunotherapy or some combination of these (Gradishar et al., 2020). Although these treatments improve survival, breast cancer patients face many challenges during treatment, including the physical impacts of the disease, its treatment, and treatment side effects as well as psychological and social impacts of the cancer experience such as loss of hope and a sense of lost control over life (Hsu et al., 2017; Li et al., 2018). These experiences have strong associations with body image distress (Rezaei et al., 2016).

For patients, the most difficult phase of cancer treatment is usually the first year after a diagnosis (Park et al., 2017), in which patients experience the diagnosis, symptoms, treatment, and side effects. Breast cancer and its treatment are highly distressing because of their potentially large impacts on body image. Promoting resilience is an essential component of psychological and social care for these patients, and the role of resilience in cancer recovery has recently attracted the attention of researchers. For example, researchers have established a significant positive association between resilience and quality of life (QOL) in breast cancer patients (Zhang et al., 2017). Factors that reportedly contribute to resilience in diverse cancer populations include social support and hope (Li et al., 2016). Each phase of the cancer experience profoundly affects the life of the patient, and the role of resilience differs in each phase. Therefore, the current study investigated the association between body image distress and resilience in breast cancer patients undergoing treatment as well as the roles of hope and social support in this association.

### Body Image Distress

Body image can be defined as a mental image of one's body as well as an attitude about one's appearance, state of health, and sexual functioning (Rezaei et al., 2016). Thus, a negative body image can cause body image distress. Surgery can negatively affect body image by causing physical changes such as post-operative scarring, swelling, redness or lymphedema. Tumors, scars, and disfigurement can contribute to body image distress by causing a loss of identity and a sense of lost control over the body (Yamani Ardakani et al., 2020). Additionally, chemotherapy and hormone therapy can cause body changes such as hair loss, weight gain, vaginal dryness, etc. These changes can diminish self-perceived sexual attractiveness, libido, and even fertility (Kołodziejczyk and Pawłowski, 2019). Patients may also experience emotional distress caused by a sense of lost control over their bodies, impaired body image, and the fear of cancer recurrence (Yamani Ardakani et al., 2020). In breast cancer patients, body image distress has been linked to late cancer stage and increased time since diagnosis (Mcclelland et al., 2015). Therefore, we hypothesized the following:

Hypothesis 1 (H1): Cancer stage is significantly associated with body image distress.
Hypothesis 2 (H2): Time since diagnosis is significantly associated with body image distress.

### Resilience

Resilience can be defined as the ability of an individual to maintain or restore relatively stable psychological and physical functioning even when living under adverse conditions or circumstances (Seiler and Jenewein, 2019). That is, resilience is not a personality trait, but a dynamic process in which life changes motivate an individual to restore balance or establish a new balance in life and to evolve positively. Such changes can include changes in life circumstances, in the environment, and in situational or contextual factors (Sisto et al., 2019). For an individual with high resilience, these destabilizing life changes can have positive outcomes by providing opportunities for in-depth self-reflection and opportunities to redefine the self through positive changes in self-perception, outlook, and emotional stability. The insight gained from this experience and the search for inner resources needed to address and overcome these challenges further reinforce the features of resilience. Consequently, individuals with high resilience actively apply adaptive strategies, e.g., seeking social support, that help them cope with and overcome adversity and restore life balance (Rabenu and Tziner, 2016; Sisto et al., 2019). This study defined resilience as the process of adapting to difficult life circumstances.

Therefore, promoting resilience is an essential component of psychological and social care for breast cancer patients. Cancer patients with strong resilience have the positive attitude and internal strength needed to cope with the disease and its treatment. Patients with high resilience tend to have a positive emotionality, a sense of purpose in life, spirituality, and ability to find a life meaning. Factors that affect resilience include individual factors, family factors, and environmental factors. According to the resilience model developed by Kumpfer (Kumpfer, 1999), the overall resilience of an individual depends on the balance between risk factors and protective factors against low resilience. In adverse life circumstances, highly resilient individuals exhibit positive adaptation behaviors that maximize protective factors and minimize risk factors (Kumpfer, 1999). Risk factors for low resilience in cancer patients include the stress caused by the disease and its treatment as well as emotional and psychological distress such as body image distress. Protective factors in the resilience of these patients include social support, and hope. Women rely on various internal resources (e.g., hope) and external resources (e.g., social support) to cope with their breast cancer.

### Hope

Hope is defined as a positive expectation of a good future. Hope is a complex multifaceted motive for life and a prerequisite for effective coping and decision-making (Ye et al., 2018). For breast cancer patients, hope is an important quality because it provides the internal strength needed to fight with the disease (Li et al., 2018). Notably, hope protects cancer patients against physical and mental stress by giving meaning to the cancer experience and by giving a reason for survival. Thus, hope is a positive psychological resource that helps patients adapt to cancer and helps them maintain and improve their well-being and QOL (Seiler and Jenewein, 2019).

### Social Support

Social support, which is a subjective perception of meaningful caring and concern in others, also promotes the formation of resilience and gives individuals the courage to face adversity, which improves their adaptability and QOL (Spatuzzi et al., 2016). Cohen and Syme (1985) identified four forms of social support from family and friends: 1) emotional support, i.e., care and support that induces trust and a sense of belonging and love; 2) esteem support, i.e., support that increases self-esteem; 3) information support, i.e., knowledge, information and advice; and 4) tangible support, i.e., financial assistance, material goods, or services (Cohen and Syme, 1985). Support from health professionals, family and friends reportedly protects against poor body image (Cohen and Syme, 1985).

### Relationship Between Body Image Distress and Resilience

Body image distress has been negatively linked to resilience in cancer patients. For example, Ristevska-Dimitrovska et al. (2015) surveyed resilience and quality of life in 218 patients (average age, 60.2 years) who had received treatment for breast cancer. Their results revealed that poor body image was associated with low resilience and poor quality of life. Another review of 12 qualitative studies in Sun et al. (2018) revealed that, in breast cancer patients, loss of the breasts and the perceived loss of integrity of the body structure caused a loss of the sense of overall harmony and symmetry of the body (Sun et al., 2018). Therefore, we hypothesized the following:

Hypothesis 3(H3): Body image distress has a significant negative association with resilience.

### Mediating Role of Hope in The Relationship Between Body Image Distress and Resilience

Body image distress is well-documented in patients with breast cancer (Rezaei et al., 2016), but little is known about the link between body image distress and hope in the breast cancer context (Liu et al., 2017; Todorov et al., 2019). Hope provides the internal strength needed to maintain emotional stability and a positive outlook while undergoing breast cancer treatment, which is often painful and disfiguring (Hatamipour et al., 2015). Studies of cancer patients have identified a strong positive relationship between body image distress and emotional distress (Liu et al., 2017; Li et al., 2018) and have identified protective effects of hope against emotional distress, including anxiety and depression (Peh et al., 2017). Hope also provides a buffer against the stress of the cancer experience and its negative impacts (Todorov et al., 2019). For cancer patients under acute and chronic stress, hope is an essential internal resource because it increases resilience, which then improves quality of life (Li et al., 2016; Solano et al., 2016). Thus, the literature suggests that hope confers a protective effect in cancer patients by reducing body image distress and by increasing resilience. Therefore, we hypothesized the following:

Hypothesis 4(H4): Body image distress has a significant negative association with hope.
Hypothesis 5(H5): Hope has a significant positive association with resilience.
Hypothesis 6(H6): Hope mediates the association between body image distress and resilience.

### Mediating Role of Social Support in the Relationship Between Body Image Distress and Resilience

In breast cancer patients, body image distress has been linked to lack of social support (Spatuzzi et al., 2016). Social support is an important external resource for coping with breast cancer. Specifically, support from health professionals, family and friends has important protective effects against body image distress. Lugton (1997) interviewed 29 women with breast cancer and found that social support reduced their stress by making the cancer threat seem less overwhelming. Specifically, social support helped them to address the challenges of living with breast cancer by enabling them to accept identity changes, uncertainty about the future, and mortality. Studies also show that social support increases hope in women who receive a new diagnosis of breast cancer after undergoing mastectomy (Denewer et al., 2011). Cancer patients who have strong social support can effectively manage the distress of body image changes (Spatuzzi et al., 2016) and tend to have high resilience (Alizadeh et al., 2018). Therefore, we hypothesized the following:

Hypothesis 7(H7): Body image distress has a significant negative association with social support.
Hypothesis 8 (H8): Social support has a significant positive association with resilience.
Hypothesis 9(H9): Social support mediates the association between body image distress and resilience.

### Relationship Between Social Support and Hope

In patients with high hope, social support reportedly exerts a positive effect on resilience. For example, low severity of symptoms and high hope were positively associated with resilience in 204 South Korea breast cancer patients undergoing chemotherapy (Yang and Kim, 2016). Although social support did not directly influence resilience, patients with strong social support tended to have decreased severity of symptoms and increased hope. Therefore, the authors inferred that social support indirectly influences resilience through hope. Another study by Ye et al. (2018) performed a questionnaire survey of resilience in 342 Chinese women undergoing breast cancer treatment. Although both hope and social support positively affected resilience, hope had a direct effect on resilience whereas social support had an indirect effect (Ye et al., 2018). Additionally, in a qualitative study by Bergqvist and Strang (2019), interviews with 20 breast cancer patients found that two forms of social support were important sources of hope: patient-doctor communication about treatment and patient-family interaction. For these patients, detailed information about their cancer treatment and reassurance that they would continue to receive treatment were essential for hope. Hope tends to be high in patients who refuse to be defined by their disease, e.g., patients who continue to participate in their daily life activities and who are willing to discuss matters other than their illness with family and friends maintaining relationships and continuing daily life activities provide the life meaning needed to maintain hope (Bergqvist and Strang, 2019). Therefore, we hypothesized the following:

Hypothesis 10 (H10): Social support has a significant positive association with hope.

In a review of the literature on body image distress and resilience in women with breast cancer, Rezaei et al. (2016) retrieved 14 relevant articles published during 1993-2016. According to their review, age and education were related to body image distress in women with breast cancer. Additionally, young age and high education level revealed strong relationships with high resilience in women with breast cancer (Wu et al., 2016; Seiler and Jenewein, 2019). Since age and education correlate with body image distress and resilience, our hypothesized model included both age and education as controlled variables.

**Figure 1:**
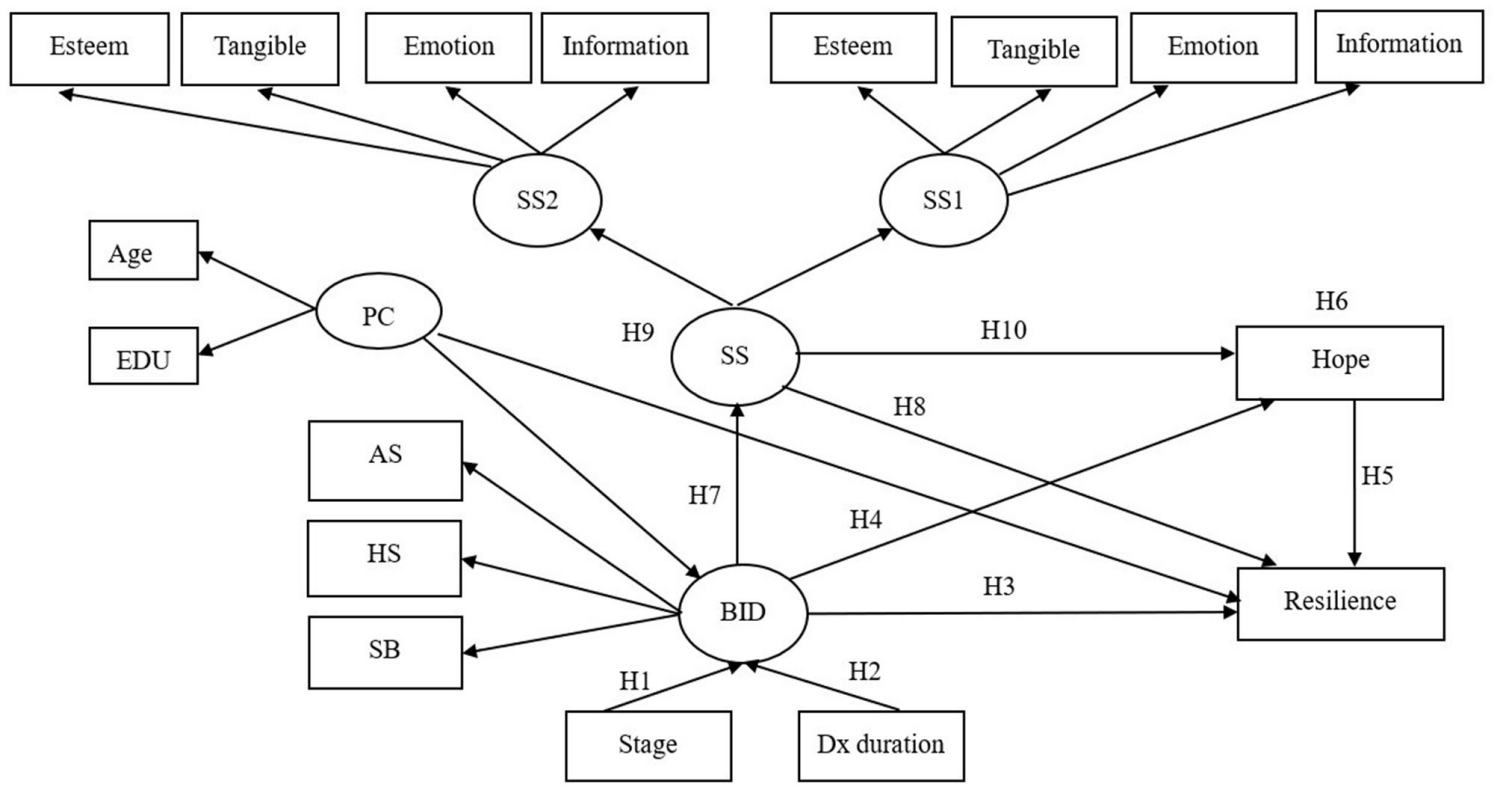
Conceptual framework of hypothesized model. PC, personal characteristic; EDU, education; BID, body image distress; SB, social barriers; AS, physical appearance and sexual life; HS, Health and strength; Stage, disease stage; Dx duration, time since breast cancer diagnosis; SS, social support; SS1, social support from family and friends; SS2, social support from health professionals; 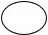 = latent variable; 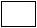 = measured variable; → = unidirectional path.

Mediating effects are conferred by intervening variables or mechanisms that transmit the effects of antecedent variables (e.g., body image distress) to outcomes (e.g., resilience) (Aguinis et al., 2017). Baron and Kenny (1986) observed that, in the stimulus-organism-response model proposed by Woodworth (1928), “an active organism intervenes between stimulus and response” and is “perhaps the most generic for stimulation of a mediation hypothesis” (p. 1176). The mediator variable is the middle variable between an independent variable (IV) and a dependent variable (DV). The purpose of including a mediator variable is to explain the relationship between an IV and a DV, e.g., to explain the relationship between a stimulus and a response. Whereas a moderator variable affects the strength and direction of this relationship, a mediator variable explains the process through which two variables are related, i.e., a mediator variable represents the generative mechanism through which the focal independent variable is able to influence the dependent variable of interest (Baron and Kenny, 1986). However, no studies have investigated the mediating roles of social support and hope in the relationship between body image distress and resilience in breast cancer patients currently undergoing treatment. Previous studies of resilience in cancer patients have investigated resilience at the time of a new diagnosis, 1 week after initiation of treatment, or after completion of treatment (Eicher et al., 2015). Therefore, the objectives of this study were to investigate mediating roles of social support and hope in the relationship between body image distress and resilience during the first year of treatment after a breast cancer diagnosis. Clarifying these mediating roles would provide medical personnel with guidelines for developing appropriate and effective interventions for increasing resilience. Figure 1 presents the conceptual model developed and tested in this study, which was based on the resilience model developed by Kumpfer (1999).

## Materials and Methods

### Participants and Setting

This cross-sectional descriptive correlation study was performed from March, 2017, to November, 2018. Factors in resilience were investigated in a convenience sample of breast cancer patients currently undergoing treatment. The recruitment sites were one outpatient oncology/ infusion department of a Medical Center and two outpatient clinics of regional hospitals in southern Taiwan. The inclusion criteria were (1) diagnosis of stage 0 to IV female breast cancer within the past 1 year; (2) current cancer treatment, i.e., chemotherapy, hormone therapy, targeted therapy, immunotherapy, bone metastasis treatment or some combination of these; and (3) ability to communicate in Mandarin Chinese. The exclusion criterion was any psychiatric or addictive disorder.

Out of 160 patients who were contacted for the study and were asked to participate, six did not complete the questionnaires due to time constraints, three declined because they felt “overwhelmed” by their cancer diagnoses, and 10 did not meet the criterion of current treatment. A “complete” questionnaire was defined as a questionnaire in which at least 80% of questions had been answered. Consent and completed questionnaires were obtained from 141 subjects (response rate = 88.1%).

The study protocol was reviewed and approved by the institutional review board of the participating hospital [KMUHIRB-E(I)-20170055], and the administrative director of each recruitment site gave approval before data collection. The researchers attended the cancer centers or outpatient clinics daily and met with eligible patients in a private room. After explaining the study objectives and methods to patients that met the enrolment criteria, the researchers obtained their consent, collected signed informed consent forms, and then asked the patients to complete a structured questionnaire. The patients were also assured that participation in the study would not affect their rights to receive medical care. Each participant completed hard copies of the demographic and structured questionnaires on the same day they gave consent to participate. The data collection procedure took 15–20 min to complete.

### Materials

Body Image and Relationships Scale (BIRS). Hormes et al. (2008) developed the 32-item BIRS by conducting focus group interviews with female breast cancer survivors. The questionnaire has three dimensions: a 12-item “health and strength” dimension for assessing perceived physical impairment related to treatment (including perceived loss of energy, health, and strength and perceived loss of control over health and strength; an example of the questionnaire items in this dimension is, “I feel physically powerful”), a 9-item “social barriers” dimension for assessing perceived impairment in social interactions (including perceived impairment of social interaction and social activity caused by embarrassment about physical or psychological symptoms; an example of the questionnaire items in this dimension is, “My concerns about my physical appearance limit my social activities”), and an 11-item “physical appearance and sexual life” dimension for assessing satisfaction with perceived changes in physical appearance and with sexual activity (including decreased enjoyment of and satisfaction with sexual activity, embarrassment about physical appearance, and altered perception that the body is “whole” and “natural”; an example of the questionnaire items in this dimension is, “I feel sexually attractive”). Each item is rated on a 5–point Likert scale from 1 point (strongly disagree) to 5 points (strongly agree). The total score ranges from 32 to 160 points, and a higher score indicates greater impairment of body image (Hormes et al., 2008). In factor analysis, the 32-item BIRS had acceptable results for the Bartlett Test of Sphericity (χ^2^ = 2361.04; *p* < 0.001). In reliability tests, the overall scale had a Cronbach α-value of 0.94 and a test-retest reliability correlation coefficient of 0.41–0.80 (Hormes et al., 2008). In the BIRS-C used in this study, the Cronbach α-values for health and strength, social barriers, and physical appearance and sexual life were 0.80, 0.91, and 0.84, respectively.

Herth Hope Index (HHI). The HHI (Herth, 1992) has 12 questions, and questions 3 and 6 are reverse-scored. Examples of questionnaire items are, “I look forward to the future,” “I have a faith that gives me comfort,” and “I feel time heals.” Each item is answered on a Likert-type scale from 1 (“strongly disagree”) to 4 (“strongly agree”). The score range is 12–48, and a high score indicates a high degree of hope. The good reliability and validity of the 12-item HHI have been established in numerous studies (Mahendran et al., 2016). The Cronbach α-values for the Chinese version of the 12-item HHI were 0.87 in Zhang et al. (2010) and 0.89 in this study.

Social Support Scale (SSS). The Chinese version of the SSS contains 31 questions for evaluating social support as perceived by breast cancer patients in Taiwan. It measures social support from family members (19 items) and from health professionals (22 items). The four SSS subscales are emotion (5 items, e.g., “They care about my breast cancer”), esteem (5 items, e.g., “They make me feel important”), information (4 items, e.g., “They take the initiative to remind me of precautions for breast cancer self-care”), and tangible support (5 items for family support and 7 items for health professional support, e.g., “When I am unwell, they give me the assistance and care I need”). Each item is answered using a 5-point Likert scale from 0 to 4. The total score ranges from 0 to76 points for family members and 0 to 88 points for health professionals, and a higher score indicates higher perceived social support. The Cronbach alpha value for the overall reliability of the SSS was 0.97 in Chu (2010) and 0.95 in the current study. In this study, the Cronbach α-values for the emotion, esteem, information and tangible support dimensions of the family support subscale were 0.93, 0.83, 0.88, and 0.77, respectively. The Cronbach α-values for these four dimensions of the health professional support subscale were 0.81, 0.90, 0.94, and 0.88, respectively.

Chinese Version of 14-item Simplified Resilience Scale (RS-14). The Chinese version of the RS-14 developed by Wagnild (2009) and translated by Tian and Hong (2013) was used to survey the resilience of the participants in this study. Each item is rated on a 7-point Likert scale from 1 (strongly disagree) to 7 (strongly agree). Examples of questionnaire items are, “I usually manage one way or another,” “My life has meaning,” “When I'm in a difficult situation, I can usually find my way out of it,” etc. The total score ranges from 14 to 98 points with a higher score indicating greater resilience. The RS-14 classifies resilience into 6 levels: very low (14–56 points), low (57–64 points), moderately low (65–73 points), moderate (74–81 points), moderately high (82–90 points) and high (91–98 points). In reliability tests of the RS-14 in Chinese cancer patients, the scale had a Cronbach α of 0.93 and a test-retest reliability correlation coefficient of 0.82 (Tian and Hong, 2013). The Cronbach alpha value for the overall reliability of the RS-14 was 0.94 in this study.

### Statistical Analysis

Statistical analysis was performed using SPSS software (version 26.0, IBM Corp., Armonk). A *p*-value < 0.05 was considered statistically significant. For demographic characteristics and disease characteristics, categorical variables were presented as frequency and percentage whereas continuous variables were described as the mean and standard deviation for each questionnaire. Analysis of Variance (ANOVA) was used to determine whether body image distress differed by cancer stage. Spearman correlation and Pearson correlation analysis were used to investigate relationships among time since diagnosis, body image distress, social support (from health professionals and from family members), hope and resilience.

The overall fit of the hypothesized model was tested by structural equation modeling (SEM), which is considered reasonably reliable for models with 75 to 200 cases (Bollen and Long, 1993). For data analysis and hypothesis testing, IBM-SPSS-AMOS package 25.0 was used in two phases: a measurement phase and a structural phase. The factorial structures of the BIRS (3 subscales) and the SSS (4 dimensions, 2 subscales) were verified in confirmatory factor analysis (CFA). In CFA of the initial measurement model, five latent factors (personal characteristics, social support, social support from family and friends, social support from health professionals, and body image distress) of 13 indicators and four measured variables were allowed to covary. Maximum likelihood method was used for data fitting. The recommended cutoffs for a good model fit are χ^2^/degree of freedom(df) < 3, goodness-of-fit index (GFI) > 0.9, adjusted goodness-of-fit index (AGFI) > 0.9, normed-fit index (NFI) > 0.9, comparative fit index (CFI) > 0.9 and root mean square error of approximation (RMSEA) < 0.05 (Schermelleh-Engel et al., 2003). If the model did not meet the recommended cut-offs for a good fit, maximum modification indices were used to adjust the fit to the ideal indices (Whittaker, 2012). Structural relationships among variables were tested as established in the theoretical model (Figure 1). Bootstrapping, which is already implemented in SEM software, was based on 2,000 resamples and was used in model fitting to determine each of the total direct and indirect path parameters and their standard errors (Leth-Steensen and Gallitto, 2016). Bias-corrected bootstrapped confidence intervals for both total and specific indirect effects within such models were also obtained (Leth-Steensen and Gallitto, 2016). If zero is not between the lower and upper bound, it can be assumed with 95% confidence to have a significant total effect or specific indirect effect.

## Results

The data analysis included 141 valid and complete questionnaires. Table 1 shows the relevant demographic and disease characteristics. The mean scores for BIRS, HHI, SSS (family), SSS (health professionals), and RS-14 were 81.62 (±16.36), 37.40 (±4.68), 59.16 (±13.85), 63.15 (±18.18), 70.86 (±13.82), respectively. The patients in this study generally revealed moderate scores for body image distress, hope, and social support and moderate-to-low scores for resilience. Differences or associations among these factors were identified by ANOVA or by Spearman or Pearson correlational analysis (Table 2). Resilience had a significant negative association with body image distress (*r* = −0.50, *p* < 0.001) and significant positive associations with education (*r* = 0.18, *p* = 0.035), hope (*r* = 0.66, *p* < 0.001), family social support (*r* = 0.28, *p* = 0.001), and health professional support (*r* = 0.26, *p* = 0.002). Resilience was not significantly associated with age (*r* =-0.071, *p* = 0.406). Hope had significant positive associations with health professional support (*r* = 0.295, *p* < 0.001) and family social support (*r* = 0. 285, *p* = 0.001) but had a significant negative association with body image distress (*r* = −0.402, *p* < 0.001). Body image distress had significant negative associations with education (*r* = −0.26, *p* = 0.002), health professional support (*r* = −0.099, *p* < 0.001) and family social support (*r* = −0.166, *p* = 0.05). Body image distress did not significantly differ by cancer stage (*F*_4.136, 0.05_ = 1.406, *p* = 0.235). Finally, body image distress was not significantly associated with age (*r* = 0.16, *p* = 0.062) or with time since cancer diagnosis (*r* = 0.12, *p* = 0.144).

**Table 1:** Demographic and disease characteristics of subjects (*n* = 141).

**Characteristic**	**Subgroups**	**Mean ± SD**	***n***	**%**
Age		53.61 ± 10.27	141	
Time (days) since cancer diagnosis		39.66 ± 43.33		
Age	30–39		15	10.6
	40–49		34	24.1
	50–59		54	38.3
	60–69		30	21.3
	70 or more		8	5.7
Education level	Lower than junior high school (lower than grade 6)		10	7.1
	Junior high school (grades 7–9)		19	13.5
	High school (grades 10–12)		52	36.9
	University/college		45	31.9
	Graduate school		15	10.6
Marital status	Unmarried		27	19.1
	Married		94	66.7
	Separated, divorced, or widowed		20	14.2
Religion	None		32	22.7
	Buddhist		62	44.0
	Christian or Catholic		23	16.3
	Taoist		22	15.6
	Other		2	1.4
Employment	Unemployed		46	32.6
	Employed full time		58	41.1
	Employed part time		12	8.5
	Retired		25	17.7
Average monthly income	< NT$20,000 (< US$625)		18	12.8
(NT$^a^)	NT$20,000~39,999 (US$625~1249)		45	31.9
	NT$40,000~59,999 (US$1250~1874)		25	17.7
	NT$60,000~79,999 (US$1875~2499)		16	11.3
	NT$80,000~99,999 (US$2500~3125)		19	13.5
	>NT$100,000 (>US$3125		18	12.8
**Cancer stage**				
	*In situ* and I		74	52.4
	II		49	34.8
	III and IV		18	12.8
Surgery type	MRM^b^		3	2.1
	TM^c^		32	22.7
	SSM^d^		2	1.4
	NPSSM^e^		31	22.0
	Partial M^f^ (formerly BCS^g^)		65	46.1
	None		8	5.7
Treatment type	ct^h^		7	5.0
	rt^i^		1	0.7
	ht^j^		97	68.8
	tt^k^		7	5.0
	ct + tt		5	3.5
	ct + ht		2	1.4
	ht + tt		5	3.5
	rt + ht		10	7.1
	Zometa (bone metastasis)		1	0.7
	ht + Zometa (bone metastasis)		2	1.4
	ht + Xgeva (bone metastasis)		1	0.7
	ct + rt		1	0.7
	ct + ht + tt		1	0.7
	tt + Xgeva (bone metastasis)		1	0.7

a *The New Taiwan Dollar (NT$) is the official currency used in Taiwan. The average exchange rate in year 2019 was US$1 = NT$31*.

b *Modified radical mastectomy*.

c *Total mastectomy*.

d *Skin-sparing mastectomy*.

e *Nipple sparing mastectomy*.

f *Partial mastectomy*.

g *Breast-conserving surgery*.

h *Chemotherapy*.

i *Radiotherapy*.

j *Hormone therapy*.

k *Targeted therapy*.

**Table 2:** Spearman's/Pearson's correlations between the study variables.

**Variables**	**1**	**2**	**3**	**4**	**5**	**6**	**7**	**8**
1. Education	1.00							
2. Time since diagnosis	−0.54^**^	1.00						
3. Age	−0.21^*^	0.27^**^	1.00					
4. Body image distress	−0.26^**^	0.12	0.16	1.00				
5. Family SS	0.09	−0.04	0.01	−0.17^*^	1.00			
6. Health professional SS	0.02	−0.08	0.14	−0.10	0.55^**^	1.00		
7. Hope	0.05	−0.12	−0.09	−0.40^**^	0.29^**^	0.30^**^	1.00	
8. Resilience	0.18^*^	−0.17^*^	−0.07	−0.50^**^	0.28^**^	0.26^**^	0.66^**^	1.00

*SS, social support;*

* *p < 0.05;*

** *p < 0.01*.

### SEM Analysis

#### Measurement Phase

In the initial measurement model, CFA was used to verify the factor structures of the SSS and BIRS. In the original SSS, factor loading exceeded 0.4 within each dimension. However, the SSS did not meet the criteria for a good model fit (χ^2^/df = 4.794, GFI = 0.886, AGFI = 0.783, RMSEA = 0.165). Maximum modification indices were used to adjust the fit to the ideal indices. For the best fit of the CFA model, “information support” and “esteem support” were dropped from SSS results for the family and friend's domain, and “esteem support” and “tangible support” were dropped from SSS results for the health professional's domain (χ^2^/df = 1.382, GFI = 0.995, AGFI = 0.951, RMSEA =0.050). Factor loadings exceeded 0.4 in three BIRS domains: health and strength (0.93), social barriers (0.67), and physical appearance and sexual life (0.81). The BIRS data had a good fit to the original model (χ^2^/df = 1.210, GFI = 0.941, AGFI = 0.905, RMSEA =0.039).

#### Structural Phase

**Figure 2:**
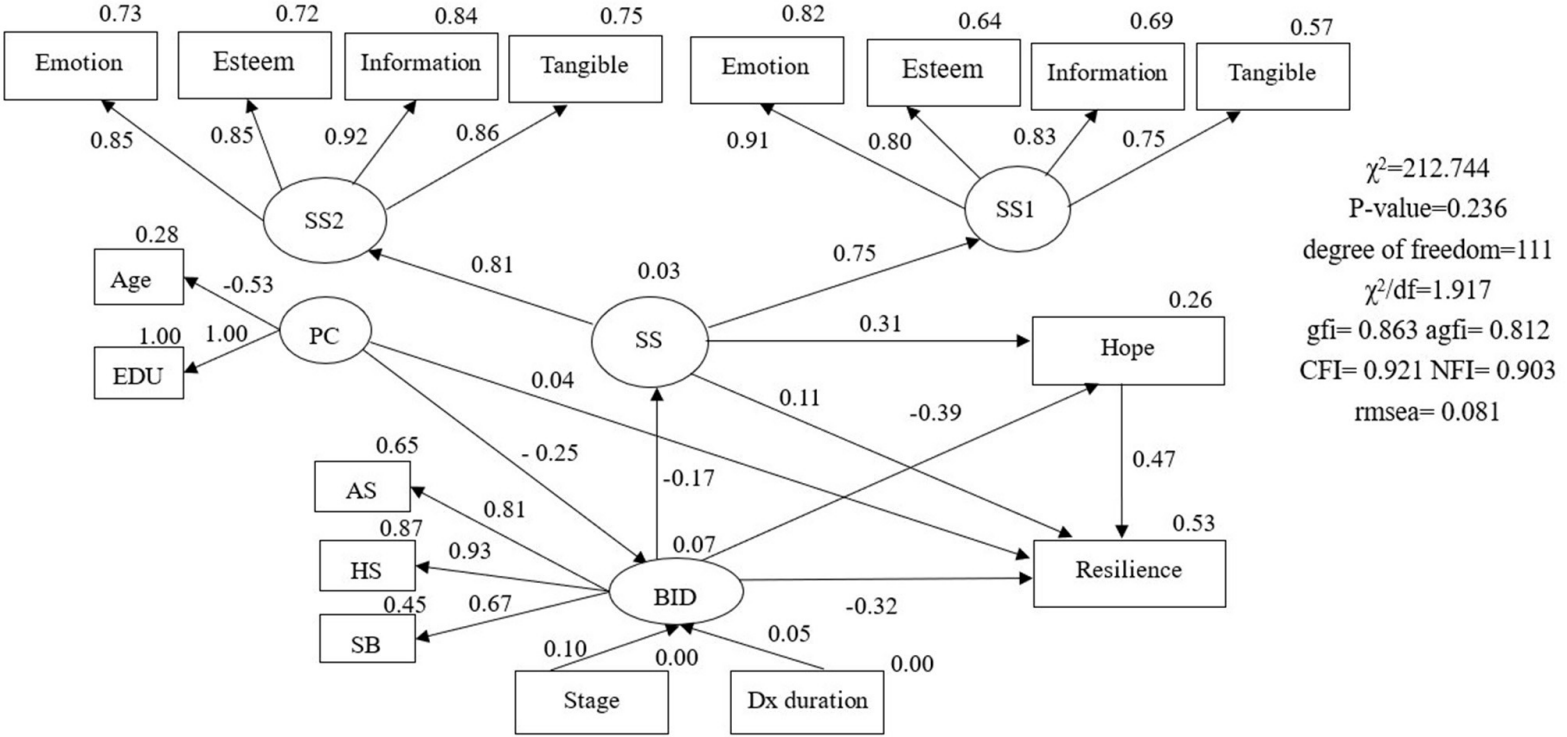
Hypothesized model with standardized estimates. PC, personal characteristic; EDU, education; BID, body image distress; SB, social barriers; AS, physical appearance and sexual life; HS, health and strength; Stage, disease stage; Dx duration, time since breast cancer diagnosis; SS, social support; SS1, social support from family and friends; SS2, social support from health professionals; 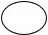 = latent variable; 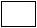 = measured variable; → = unidirectional path; *p < 0.05; **p < 0.01; ***p < 0.001.

**Figure 3:**
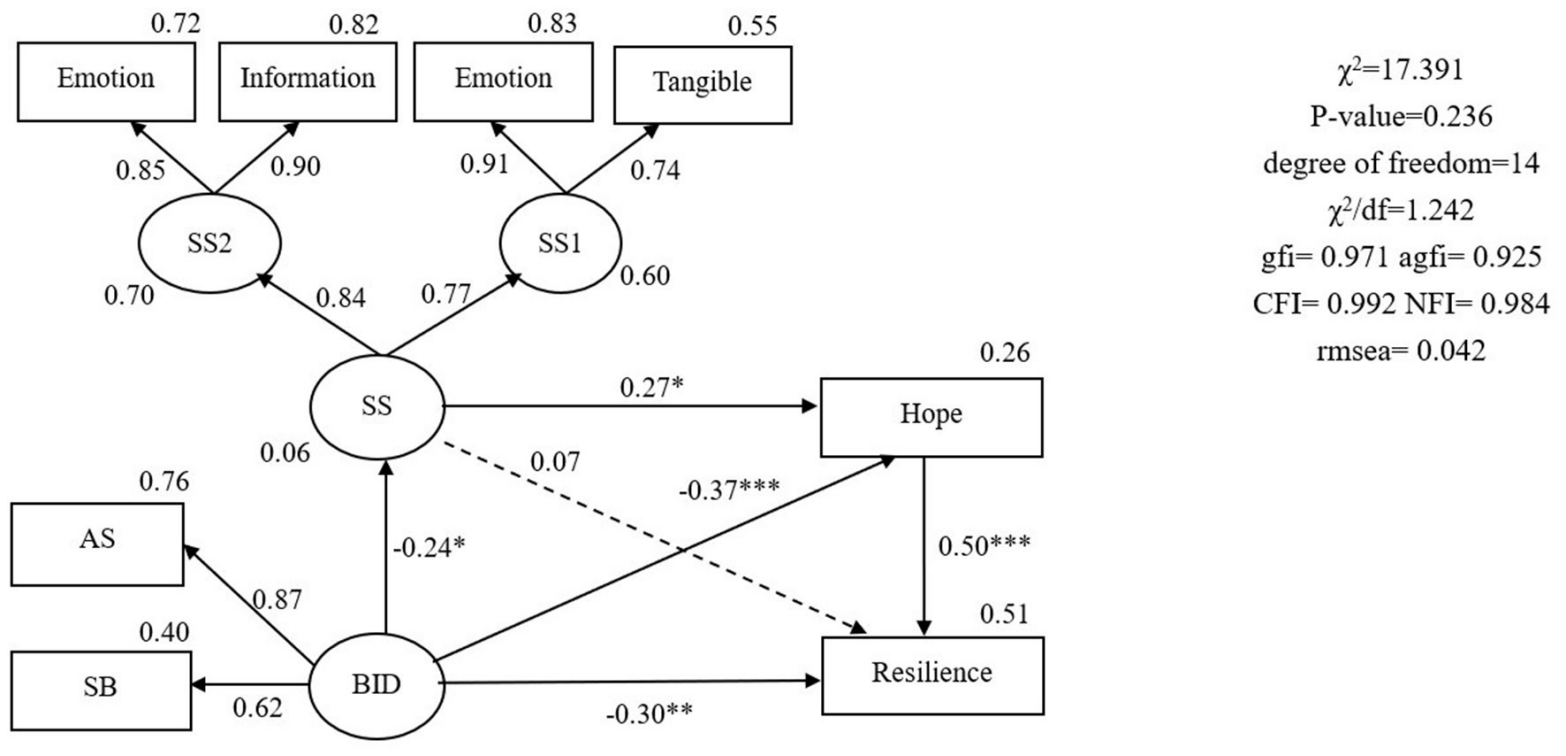
The final model with standardized estimates. BID, body image distress; SB, social barriers; AS, physical appearance and sexual life; SS, social support; SSl, social support from family and friends; SS2, social support from health professionals; 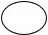 = latent variable; 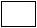 = measured variable; → = unidirectional path; *p < 0.05; **p < 0.01; ***p < 0.001.

The original hypothesized model had a poor fit to the data (χ^2^/degree of freedom = 1.917, GFI = 0.863, AGFI = 0.812, NFI = 0.903, CFI = 0.921, RMSEA = 0.081) (Figure 2). Therefore, maximum modification indices were used to adjust the fit to the ideal indices. The “age,” “education,” “stage of cancer,” “time since diagnosis,” and “health and strength” domains of body image were removed from the model. Next, the fit analysis of the revised structural equation model confirmed a good data fit (χ^2^/df = 1.242, GFI = 0.971, AGFI = 0.925, NFI = 0.984, CFI = 0.992, RMSEA = 0.042) (Figure 3). The body image scale retained two dimensions: “social barriers” and “physical appearance and sexual life.” Figure 3 presents the structural relationships and standardized coefficients, which show that significant paths identified in the analysis included paths from body image distress to hope (β = −0.37, *p* < 0.001), from hope to resilience (β = 0.50, *p* < 0.001), from body image distress to social support (β = −0.24, *p* = 0.046), from social support to hope (β = 0.27, *p* = 0.011), and from body image distress to resilience (β = −0.30, *p* = 0.002). The bias-corrected bootstrapping results further revealed that hope had a partial mediating effect on the relationship between body image distress and resilience (95% CI: −1.019 to −0.161, with a point estimate of −0.497, *p* = 0.002) (Table 3). Social support had a partial mediating effect on the relationship between body image distress and hope (95% CI: −0.181 to −0.001, with a point estimate of −0.059). Social support indirectly affected resilience through hope in the absence of an association between social support and resilience (95% CI: −0.235 to 0.029, with a point estimate of −0.045, *p* = 0.172). Therefore, another full mediating pathway was from body image distress to social support to hope to resilience (95% CI: −0.315 to −0.006, with a point estimate of −0.088, *p* = 0.041) (Table 3). In the final model, body image distress, hope, and social support accounted for 51% of the total variance in resilience.

**Table 3:** Direct and indirect effects of hope and social support on association between body image distress and resilience.

**Relationship**	**Point estimate**	**SE**	**95% CI**		***p***
			**Lower**	**Upper**	
**Total indirect effects**	−0.630	0.220	−1.224	−0.290	0.001
BID^a^ → Hope → Resilience	−0.497	0.208	−1.019	−0.161	0.002
BID → Support → Resilience	−0.045	0.058	−0.235	0.029	0.172
BID → Support → Hope → Resilience	−0.088	0.063	−0.315	−0.006	0.041
**Total direct effects**	2.338	0.882	0.357	3.821	0.027
BID → Hope	−0.334	0.098	−0.601	−0.075	0.004
Hope → Resilience	1.490	0.224	0.969	1.947	0.002
BID → Support	−0.161	0.081	−0.375	−0.025	0.039
Support → Resilience	0.280	0.317	−0.299	1.022	0.284
Support → Hope	0.366	0.187	0.080	0.754	0.025
BID → Resilience	−0.793	0.330	−1.568	−0.215	0.002
**Total effects**					
BID → Resilience	−1.423	0.440	−2.338	−0.559	0.002

a *Body Image Distress*.

## Discussion

This study is the first in Taiwan to analyze the mediator roles of social support and hope in the relationship between body image distress and resilience in breast cancer patients undergoing treatment. The patients in this study generally revealed moderately low resilience. In univariate analysis, body image distress had significant negative associations with resilience, hope and social support (supported H3, H4, H7). Social support had significant positive associations with hope (supported H10). Hope had a significant positive association with resilience (supported H5). In SEM analysis, hope and social support were significant mediators of the association between body image distress and resilience (supported H6 and H9). Social support had a partial mediating role in the relationship between body image distress and hope. Additionally, hope had a full mediating role in the relationship between social support and resilience. Although social support did not directly affect resilience (did not support H8), it indirectly affected resilience through hope (supported H6). Together, body image distress, hope, and social support accounted for 51% of the total variance in resilience.

Body image distress has been linked to low resilience in cancer patients. For example, a survey of resilience and QOL in 218 patients treated for breast cancer revealed that poor body image distress was associated with low resilience and poor QOL (Ristevska-Dimitrovska et al., 2015). Another review of 12 qualitative studies revealed that loss of the breasts and the perceived loss of structural integrity of the body caused a loss of the sense of overall harmony and symmetry of the body in breast cancer patients (Sun et al., 2018). Therefore, women rely on various internal and external resources to cope with breast cancer. Hope, which is an internal resource, provides the internal strength needed to fight breast cancer (Liu et al., 2017). In our patients, hope was a very important mediating factor in the relationship between body image distress and resilience, which has not been reported previously. Hope had a partial mediating effect on the relationship between body image distress and resilience, and social support indirectly affected resilience through hope. Previous studies have only reported that hope is the best predictor of resilience in breast cancer patients (e.g., Wu et al., 2016, in a study of 213 newly diagnosed breast cancer patients), which is consistent with our findings. Body image impairment is well-documented in patients with breast cancer, but little is known about the link between body image distress and hope in this context (Todorov et al., 2019). Prior works have identified a strong positive relationship between body image distress and emotional distress (Li et al., 2018). Hope is a buffer against negative and stressful events in the cancer experience. Thus, the literature suggest that hope confers a protective effect in cancer patients by reducing body image distress and by increasing resilience, which is consistent with our results.

In addition to hope, another factor that revealed a full mediating role in this study was social support, which is an external resource. Social support positively affected resilience by increasing hope. For example, a low symptom experience and high hope were positively associated with resilience in 204 South Korea breast cancer patients undergoing chemotherapy (Yang and Kim, 2016). Although social support did not directly influence resilience, it decreased symptom experience and increased hope. The authors inferred that social support indirectly influences resilience through hope (Yang and Kim, 2016). Another study by Ye et al. (2018) performed a questionnaire survey of resilience in 342 Chinese women undergoing breast cancer treatment. Hope had significant direct effects on their resilience whereas social support had significant indirect effects on their resilience.

Emotional and tangible support were retained in the family and friends support subscale of the proposed model. Family and friends mainly provide emotional support (Lugton, 1997). Support from partners and loved ones is a particularly important social support. Partners and loved ones must have the sensitivity to broach the topic at an appropriate time and manner, and the woman must feel free to discuss her anxieties. Work colleagues can give emotional support to women with breast cancer by expressing concern and by minimizing anxiety about taking time off from work (Lugton, 1997). Tangible support is physical support, e.g., assistance with household chores, cooking, bathing and other self-care activities, childcare, and even simple tasks such as noting the date of a doctor appointment (Hirschman and Bourjolly, 2005). Family and friends usually provide tangible support in their primary and secondary roles. For women with breast cancer, tangible support is usually provided by a partner or by the mother (Hirschman and Bourjolly, 2005).

Information support and emotional support were retained in the health professional support subscale of the proposed model. Women with breast cancer often seek support in the form of information about appearance, e.g., the most suitable protheses or clothing. Health professionals must consider the information needs of the patient, i.e., current knowledge related to breast cancer and its treatment, including self-care. Notably, assessment of information needs of patients with breast cancer should account for cultural factors. For example, in Nair et al. (2018), an assessment of information needs in Chinese women with breast cancer revealed an unmet need for information related to sexuality and negative body image. However, the low need for this information may have been related to the tendency to avoid discussion of issues related to sexuality and body image in Asian culture. Even if patients are willing to discuss issues of sexuality and negative body image, health professionals in Asian countries rarely provide useful information relevant to these issues because they lack skills in identifying and managing these issues. Most breast cancer patients have an ongoing need for information, which must be delivered with sensitivity, honesty, and patience. A multidisciplinary approach to addressing psychosexual issues can improve their sexual well-being, which would then enhance their QOL.

Although family and health professionals have important roles in the development of individual resilience, most studies of resilience have only investigated western populations (Eicher et al., 2015). In traditional eastern culture, ethical and philosophical systems (e.g., Confucianism) and religious systems (e.g., Buddhism) tend to emphasize the importance of family and social groups whereas analogous systems in western culture tend to emphasize the importance of the individual. Asian culture de-emphasizes the importance of the individual by encouraging self-reflection and suppression of emotional displays (Schouten et al., 2020). Cultural factors such as these should be considered when assessing body image distress, hope and resilience and when designing and implementing interventions for increasing resilience, particularly during face-to-face sessions. Additionally, cancer stage and time since diagnosis had no significant association with body image distress. However, the numbers of patients with stage III and IV breast cancer in this study were small, and time since diagnosis varied widely. The roles of cancer stage and in body image distress need further study in a larger sample.

The effectiveness of breast cancer treatment can be increased by identifying and supporting patients who are prone to high body image distress. Therefore, we suggest that, in routine clinical assessments of breast cancer patients, two dimensions of the 32-item BIRS should be administered to assess body image distress: the “social barriers” dimension and the “physical appearance and sexual life” dimension. Health professionals can also refer patients for psychological counseling or other interventions to address body image distress. Since this study also revealed that hope had a buffering effect against the negative psychological and social effects of body image distress in women with breast cancer, interventions for increasing hope should include providing resources to increase the ability to manage and cope with emotional distress, encouraging participation in social networks, and suggesting strategies for finding new meaning in life. Finally, cognitive behavioral therapy has proven effective for inducing a constructive perception of breast cancer.

Acknowledged limitations of this study are the use of convenience sampling and the somewhat small sample size for an analysis of this type, which limited the representativeness of the investigated breast cancer population. The cross-sectional design of this study also limited the ability to infer causality. Although this study provides some insight into the mediator roles of hope and social support in the association between body image distress and resilience in breast cancer patients undergoing treatment, further studies are needed to collect additional qualitative and longitudinal data in a larger cancer population.

## Summary

Hope and social support were important mediating factors in the resilience of breast cancer patients currently receiving treatment. Health professionals can improve their care quality by understanding how hope, social support, and other mediating variables affect the relationship between body image distress and resilience and by applying a conceptual framework that increases resilience by minimizing risk factors and maximizing protective factors.

## Data Availability Statement

The data that support the findings of this study are available on request from the corresponding author. The data are not publicly available due to privacy or ethical restrictions. Requests to access the datasets should be directed to hthsu@kmu.edu.tw.

## Ethics Statement

The studies involving human participants were reviewed and approved by Kaohsiung Medical University Hospital Institutional Review Board. The patients/participants provided their written informed consent to participate in this study.

## Author Contributions

H-TH and H-FH conceived and designed the study, involved in title selection, data analysis, drafting of the manuscript, and approved the final manuscript. C-HJ and H-TH analyzed the data, recruited the study participants, evaluated them, and collected the data. H-TH wrote the manuscript. H-TH and J-LC were involved in the interpretation of the data and contributed to the manuscript preparation. All authors read and approved the final manuscript.

## Funding

The authors gratefully acknowledge the supports from the Ministry of Science and Technology, Taiwan (MOST 108-2635-B-037-007- and MOST 110-2511-H-037-001-). An article processing charge (APC) is supported by the Kaohsiung Medical University, Taiwan, Grant Number (KMU-M110005).

## Conflict of Interest

The authors declare that the research was conducted in the absence of any commercial or financial relationships that could be construed as a potential conflict of interest.

## Publisher's Note

All claims expressed in this article are solely those of the authors and do not necessarily represent those of their affiliated organizations, or those of the publisher, the editors and the reviewers. Any product that may be evaluated in this article, or claim that may be made by its manufacturer, is not guaranteed or endorsed by the publisher.
